# Evaluation of prevalence and risk factors associated with *Cryptosporidium* infection in rural population of district Buner, Pakistan

**DOI:** 10.1371/journal.pone.0209188

**Published:** 2019-01-02

**Authors:** Asar Khan, Sumaira Shams, Saima Khan, Muhammad Iftikhar Khan, Sardar Khan, Abid Ali

**Affiliations:** 1 Department of Zoology, Abdul Wali Khan University Mardan, Mardan, Pakistan; 2 Centre for Biotechnology and Microbiology University of Swat, Swat, Pakistan; 3 Department of Environmental Science, University of Peshawar, Peshawar, Pakistan; Universita degli Studi di Parma, ITALY

## Abstract

**Background:**

*Cryptosporidium* spp are important intestinal protozoan parasites that cause diarrhea in humans, domestic and wild animals. Its infection remains a main public health concern however, the epidemics in human being is still unclear, particularly in developing countries. There are several factors that may enhance the spreading of this parasite in human population especially in young children.

**Methodology:**

A questionnaire was designed to obtain the demographic and clinical data from the participants. A total of 425 stool samples were collected from suspected children (aged 3–10 years) in different hospitals and villages. The initial screening was performed with modified Ziehl Neelsen (mZN) staining technique followed by polymerase chain reaction (PCR). Several potential risk factors were also assessed through the obtained information from suspected individuals.

**Results:**

Out of all 425 collected samples, 127 were observed positive by mZN with a prevalence of 29.88% (127/425). The 127 mZN positive samples together with 50 mZN negative samples were processed for molecular analysis through PCR assay. Among them, 71 out of 127 mZN positive samples and 4 out of 50 mZN negative samples were found positive by PCR. The molecular analysis showed that *Cryptosporidium parvum* was the main cause of infection in children. The results revealed that individuals exposed to diarrhea were more likely to be infected with *Cryptosporidium* infection while several environmental factors may also play a key role in spreading of this parasite.

**Conclusions/Significance of the study:**

The current high prevalence of *Cryptosporidium* infection may be due to the lack of awareness and routine based testing in identification of this parasite in District Buner. Further studies are required to determine the importance of *Cryptosporidium* infection in this area as well as across the country and to find out the possible risk factors that may be associated with the occurrence of this protozoan. There is, however, an urgent need for laboratory-based observational studies to develop a more dynamic estimate of the cryptosporidial disease burden in the region.

## Introduction

*Cryptosporidium* spp are enteric protozoan parasites of medical and veterinary importance that infect a wide range of humans and animals globally [1]. Defining species within the genus *Cryptosporidium* has also been debated and at present there are 27 species reported as valid [2]. In humans, nearly 20 *Cryptosporidium* species were detected, among them the *C*. *hominis* and *C*. *parvum* are the most reported species [3]. The spreading of these parasites occurs through fecal-oral route and also by consumption of contaminated water or food and zoonotic or anthropogenic transmission [4]. Cryptosporidiosis is usually results in watery diarrhea which might be occasionally teeming and prolonged [5]. The additional signs includes low grade temperature, nausea, intestinal pain and vomiting and occasionally anorexia, headache, malaise, myalgia and faintness [6]. The length of the oocyst ranges from 4.5 to 7.5 μm and width 4.2 to 5.7 μm and the size mainly depends on the host they infect [7].

Worldwide, the *Cryptosporidium* infection is estimated and responsible for about 30–50% of deaths in young individuals and found to be the second highest reason of diarrhea and deaths in children after rotavirus [8]. Till 2007, 325 outbreaks of parasitic protozoan disease have been reported in which the *C*. *parvum* was responsible for 50.8% of outbreaks and among them 93% outbreaks occurred in North American and European countries. Out of them, 30% of outbreaks were recorded from Europe, with the UK accounting for 24% of all global outbreaks [9]. The majority of *Cryptosporidium* outbreaks occurred in Australian continent followed by North America and Europe [10]. A recent study showing research impetus given to the monitoring of parasitic protozoan diseases from 53 countries (1970 to 2016) revealed that North America contributed 36% of the total publications followed by Europe (31%), Asia (19%), Central and South America (7%), Oceania (5%) and Africa (3%) [11].

A current epidemiological report from 3 African and 3 Asian sites confirmed that *Cryptosporidium* spp were the 2^nd^ most prevalent parasite responsible for severe diarrhea and high morbidity in youngsters (age 12–23 months) [12]. In sub-Saharan Africa, it has been estimated that over 2.9 million of *Cryptosporidium* infections occur every year in children aged less than 24 months [13]. From 1979 to 2015, sixteen outbreaks of water-borne protozoa were also reported in Latin American countries and among these outbreaks, the *Cryptosporidium* spp were the most commonly found protozoa [14].

The existing traditional diagnostic methods which are used for identification of stool parasites include microscopic analysis of stool smears. The microscopic detection of stool parasites through staining methods have high rate of parasites identification [15]. The Immunomagnetic separation (IMS) is designated as a supplementary concentration step that separate the *Cryptosporidium* spp oocysts in water samples however, due to a high cost this assay is not generally used in diagnostic laboratories for stools samples. IMS have been used with environmental samples to separate the oocysts from food, water and soil samples [16]. The molecular technique such as PCR for the laboratory diagnosis of cryptosporidiosis exhibits an outstanding specificity and sensitivity in the detection and identification of these parasites at specie level [17]. Similarly, the LAMP technique (Loop-mediated isothermal amplification) is also a molecular method that unlike traditional molecular methods is less expensive and has been developed and applied for the detection of waterborne cryptosporidiosis during the last years [18]. Moreover, several water treatment plants use the Environmental Protection Agency (EPA) method No. 1623 and more recently the modified EPA 1623.1 version. These methods are considered to function as basic protocol for the detection of *Cryptosporidium* spp in aqueous environments through concentration by filtration (using expensive filters). However, it requires highly trained laboratory staff, expensive infrastructure and equipment [19].

Pakistan is a developing country and 64% of its population lives in rural areas where diarrheal diseases remain the leading cause of children death. Taking into account, about 30.4% of the households in rural areas of Pakistan have no access to an improved sanitation [20]. According to WHO, the mortality rate due to diarrheal diseases resulted in 1.4 million deaths only in 2015. Also, studies showing the distribution of death in children under the age of 5 years with 11% of the mortality being attributed to the diarrhea [21]. Moreover, some earlier studies using staining and molecular methods on human and animal cryptosporidiosis reported a high prevalence from various parts of Pakistan such as Ravi and Patoki (73.33%), Karachi (55.0%), Sindh (53.0%), Lahore (25.6%), Skardu (20.8%) and Peshawar (9.0%) [22–27].

Such alarming mortality rate among children warrant attention towards the probable link between the provisions of unsafe drinking water, poor sanitation and the incidence of diarrheal diseases among children, particularly in rural areas. Studies about *Cryptosporidium* infection in Khyber Pakhtunkhwa (KP) state of Pakistan are limited. Therefore, present study aims to determine the prevalence of *Cryptosporidium* infection in suspected individuals and patients that are already referred to health care centers and hospitals in District Buner KP, Pakistan. Also, to find out the risk factors associated with *Cryptosporidium* infection to facilitate the awareness and health education of local population and clinicians about the importance of cryptosporidiosis testing and diagnosis on routine basis.

## Methods

### Description of the study area

**Fig 1:**
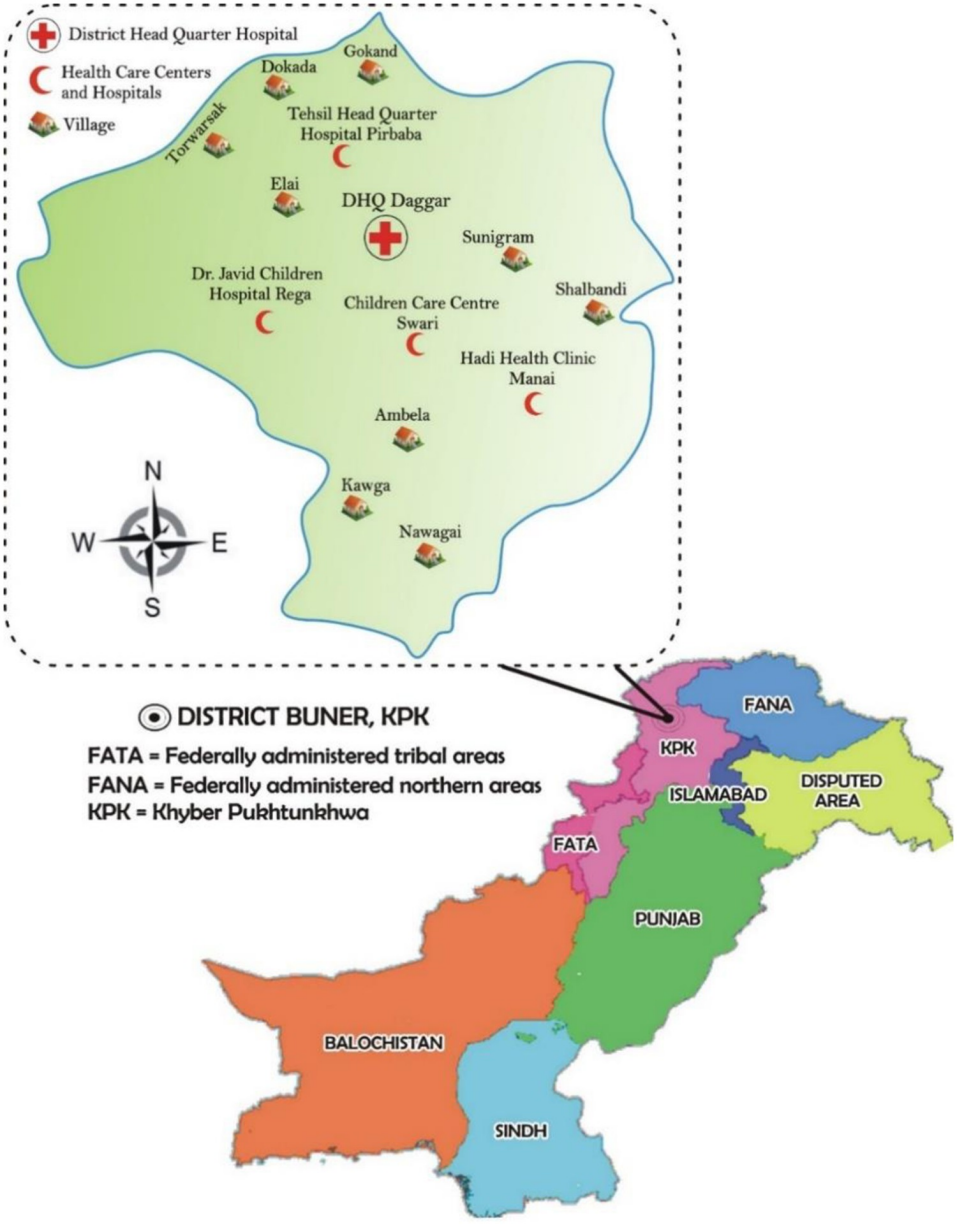
Geographical representation of District Buner (KP Pakistan), and sampling sites (highlighted).

The study was conducted in District Buner (KP, Pakistan) which lies between 34–9 and 34–43 °N latitude and 72–10 and 72–47 °E longitude. The climatic conditions of District Buner differs with the elevation and can be categorized as dry sub-tropical region where the climate remains pleasant throughout the year. During summer the Monsoon rains while in winter snowfall on the mountains peaks is common. There is a gradual raise in temperature (maximum up to 44 °C) during summer and in winter it slowly drops down to -2 °C. The average annual rainfall is approximately 30 inches. According to the Ministry of Health Bio-Statistics Section and Health Management Information System Islamabad, there are 19 BHU (Basic Health Units), 14 private hospitals and health care centers, 8 civil dispensaries, 3 RHC (Rural Health Centers) and 4 civil hospitals including DHQ Hospital (District Head Quarter) Daggar in District Buner (Fig 1).

### Ethical considerations and approval of the study

The ethical committee of Department of Zoology Abdul Wali Khan University Mardan, approved the study design and the written consent procedures. Permission was obtained from the heads of Hospitals, Health care centers and families of suspected persons. To facilitate the sampling from patients attending the hospitals and health care centers, the families of participants were also informed verbally about the purpose of the study. The data of demographic features, symptoms, economic status, source of drinking water and contact with positive animal or human were obtained through a standard questionnaire designed by the medical experts (S1 File).

### Sample collection & preservation

A total of 425 fecal samples from suspected children (aged 3 to 10 years) presenting the signs and symptoms of diarrhea were collected in District Buner during March to October 2016. Out of which, 40 samples were collected from Children Care Centre (CCC) Swari, 27 from Sunigram, 40 from Hadi Health Care Clinic (HHCC), 15 from Shalbandi, 55 from DHQ Daggar, 23 from Cheena, 31 from village Elai, 26 from Torwarsak, 14 from BHU Pir baba, 38 from Ambela, 23 from Kawga, 16 from Nawagai, 26 from Dokada and the remaining 41 samples were collected from Dr. Javid Children Hospital Rega.

All the samples were collected in sterile 5 ml tubes containing 2 ml of 75% ethanol and brought to Parasitology Lab, Abdul Wali Khan University Mardan. The staining method was performed upon receiving of the samples and the positive samples were stored at room temperature for 3 to 5 days until further analysis.

#### Modified Ziehl Neelsen (mZN) staining

The stool sample was spread evenly on the middle of the slide with constant rotational movement. The slides were placed on dryer with smeared surface upwards and air-dried for about 10 minutes. The dried smear was fixed with absolute methanol for 3–5 minutes. Carbol-Fuchsine solution was added to the slide covering the whole smear for 15–20 minutes. The slide was washed gently with tap water using a dropper. After this, 4–6 drops of decolorizer acid alcohol was added to the smear and the slide was washed off with clean water again. The counter stain methylene blue was added for 4–5 minutes and washed with water. The back side of the slide was rubbed, cleaned and put in the draining rack for 5 minutes to air dry the smear. The smear was examined microscopically, using the 40x and 100x (oil immersion lens) objectives and scanned thoroughly for parasite identification.

http://dx.doi.org/10.17504/protocols.io.sageabw [PROTOCOL DOI]

#### Identification and morphologic analysis

The morphological examination of *Cryptosporidium* spp oocyst was performed for fecal smear following the Connelly [28] identification method. Photographs for *Cryptosporidium* spp positive stained slides were recorded through Canon digital camera (16MP, Model # A-4000 IS, Japan).

#### DNA extraction and amplification

After the identification of *Cryptosporidium* positive samples, the remaining portion of each sample was measured and preserved by adding two volumes (1:2) of 75% ethanol to each tube. After thoroughly mixing, the positive samples were than subjected to PCR assay.

The genomic DNA was extracted using, GF-1 Soil sample DNA extraction kit (Vivantis Technologies, Malaysia). The given standard protocol was used for DNA extraction with some minor modifications. The extracted DNA was then subjected to thermal cycler (Bio-RAD, T100, USA) for PCR reaction using *Taq* DNA polymerase. In a 20 μl volume of PCR reaction, the mixture for single reaction was composed of 4 μl of extracted DNA, 2 μl 10X PCR buffer, 2 μl MgCl_2_, 1 μl dNTPs, 1 μl (10 μM) of forward and reverse primers, 7 μl RNase free water and 2 μl *Taq* DNA polymerase. Specific primers, forward-AWA722F 3′-AGTGCTTAA AGCAGGCAACTG-5′ and reverse-AWA1235R 5′-CGTTAACG GAATTAACCAG AC-3′(IDT, Canada) was used to amplify 18s rRNA gene (556-bp) of *C*. *parvum* [29]. The PCR assay was performed as follows: after an initial dsenaturation step of 3 minutes at 94 °C, a set of 35 cycles was run each consisting of 45 seconds at 94 °C, 45 seconds at 60 °C and 1 minute at 72 ◦C, with a final extension of 5 minutes at 72 °C. The specific amplified DNA fragment was visualized through ethidium bromide staining using UV-trans illuminator (UVP Bio-Doc, California, USA). http://dx.doi.org/10.17504/protocols.io.r9sd96e [PROTOCOL DOI]

#### Statistical and graphical presentation of data

The data was analyzed by XLSTAT-BIOMED V.19.03 (Addinsoft, NY, USA) and Graphpad Prism V.7.0 (CA, USA). For the summarization of data, the simple descriptive statistics (mean and ±SD) were used for continuous variables. The prevalence of *Cryptosporidium* infection for each level of categorical variables number (%) was used and the odd ratio was calculated with 95% confidence interval (Lower & Upper) through Baptista-Pike test. The association between categorical variables and the prevalence of *Cryptosporidium* infection was estimated by the *Fisher`s* exact test with a *p*-value of ≤ 0.05 statistically significant. Moreover, the graphical representation, cropping and editing of figures (Map) was done through Adobe Photoshop 7.0 (USA) and Coral Draw 9.0 (SYNEX, Brooklyn, NY).

## Results

### Microscopic examination

**Fig 2:**
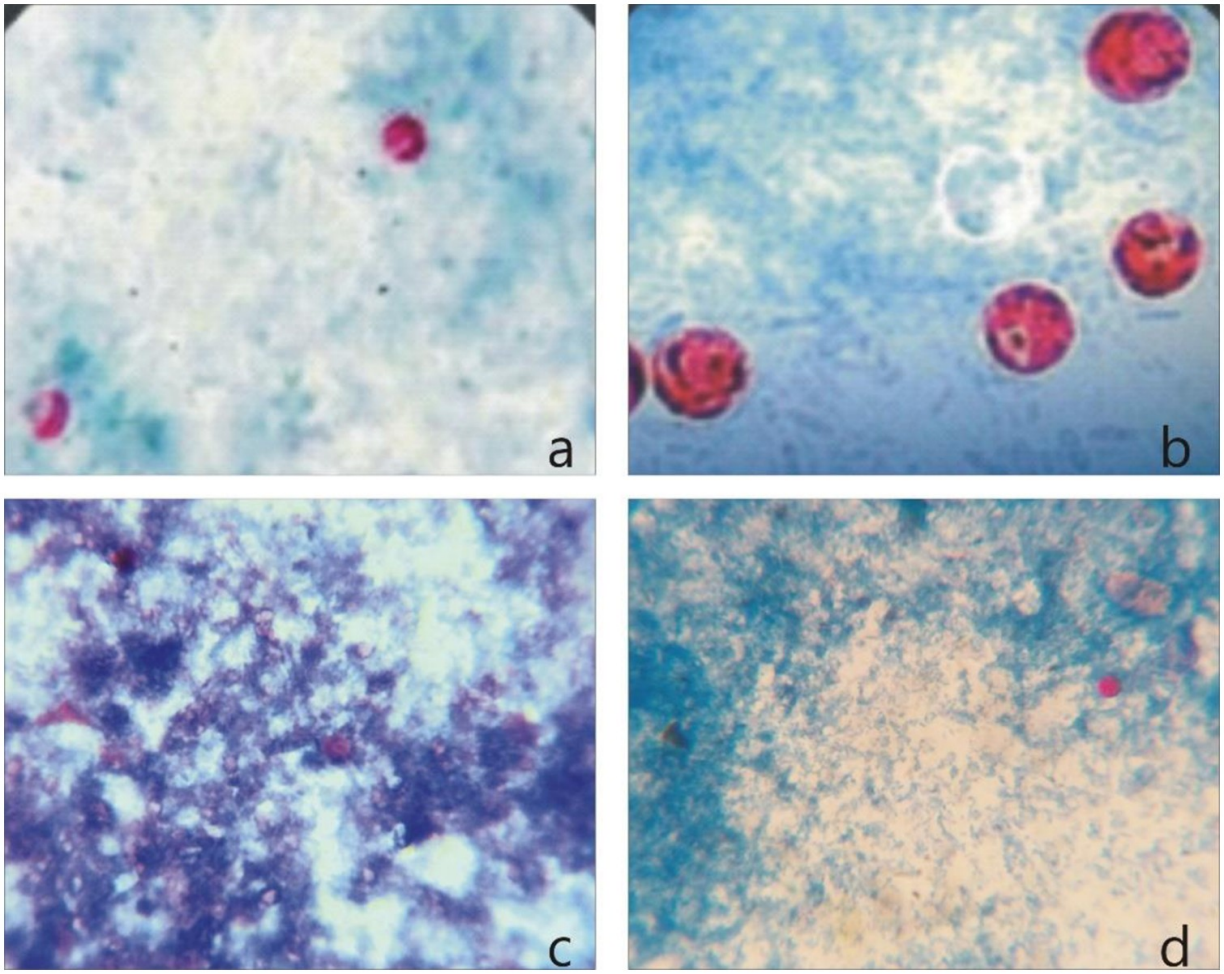
Where (a, b) shows the *Cryptosporidium* spp oocysts positive smear observed as thick walled spherical structures and stained pink-red color and (c, d) negative smear with pink stained hollow structures.

The microscopic examination of the stool samples through mZN staining showed the presence of *Cryptosporidium* spp oocysts in 127/425 (29.88%) samples from different areas of District Buner. The oocysts of *Cryptosporidium* spp observed with a sphere-shaped morphology and blurry inner structure (Fig 2).

The high prevalence of *Cryptosporidium* infection was recorded in different areas such as Dokada 42.30%, HHCC 37.50%, DHQ 36.36%, CCC 30.00% and Ambela 28.94%. Low prevalence was recorded in village Shalbandi (26.66%), Cheena (26.08%), Kawga (26.08%), Elai (22.58%), DJCH (21.95%) and Torwarsak (19.23%). The highest observed prevalence was in BHU Pirbaba (54.16%) and the lowest was observed in village Nawagai (18.75%) and Sunigram (18.51%) (Table 1).

**Table 1:** Overall prevalence (%) of *Cryptosporidium* infection among male and female (n = 425) in different demographic regions of District Buner.

Area/Location	Gender			Detection of +ve cases			% age
	Male	+ve	Female	+ve	Total Exam.	Total +ve	
Children Care Centre	25	9	15	3	40	12	30.00%
Sunigram	15	3	12	2	27	5	18.51%
Hadi Health Care Clinic	30	8	10	7	40	15	37.50%
Shalbandi	10	2	5	2	15	4	26.66%
DHQ Daggar	40	11	15	9	55	20	36.36%
Cheena	15	4	8	2	23	6	26.08%
Elai	20	2	11	5	31	7	22.58%
Torwarsak	3	2	23	3	26	5	19.23%
Basic Health Unit Pirbaba	10	7	14	6	24	13	54.16%
Ambela	25	7	13	4	38	11	28.94%
Kawga	15	4	8	2	23	6	26.08%
Nawagai	5	1	11	2	16	3	18.75%
Dokada	10	7	16	4	26	11	42.30%
Dr. Javid Children Hospital	24	4	17	5	41	9	21.95%
**Sum =**	**247**	**71**	**178**	**56**	**425**	**127**	**29.88%**

In gender-wise prevalence ratio, there was no significant difference (*p*-value = 0.38) observed among male and female individuals. A total of 247 male samples were examined out of which 71 (Mean = 5.07, SD = ±3.08) samples were found positive for parasite oocysts. On the other hand, a total of 178 female samples were screened with 56 (mean = 4.00, SD = ±2.10) positive cases. For the gender wise prevalence, the odd ratio (OR = 0.83) was calculated with 95% confidence interval (Upper = 0.55 & Lower = 1.26) which shows statistically no significance of *Cryptosporidium* spp prevalence.

### Prevalence of *Cryptosporidium* infection among diarrheic and non-diarrheic individuals

The collected samples were grouped into diarrheic (n = 330) and non-diarrheic (n = 95) for estimation of variances in prevalence between diarrheic and non-diarrheic individuals. On microscopic examination of these groups, a total of 244 samples (202 diarrheic and 42 non-diarrheic) were screened for age group 3–5 years, out of which 83 (41.0%) of diarrheic and 7 (16.6%) non-diarrheic were found positive for *Cryptosporidium* infection. For age group 6–10 years, a total of 181 samples were examined and the observed prevalence was 25.7% in diarrheal and 7.5% in non-diarrheal cases (Table 2).

**Table 2:** The demographic features of study subjects with respect to diarrheic and non-diarrheic cases (n = 425).

Age groups	Total exam.		Diarrheic cases		Non-diarrheic cases	
			No. of exam.	Positive/%	No. of exam.	Positive/%
**3–5 years**	244		202	83 (41.0%)	42	**7 (16.6%)**
**6–10 years**	181		128	33 (25.7%)	53	**4 (7.5%)**
**Total**	**425**		**330**	**116**	**95**	**11**

The data showed a significant difference (*p*-value = 0.01) among diarrheic (Mean = 58.00, SD = ±35.36) and non-diarrheic (Mean = 5.50, SD = ±2.12) individuals which suggest that individuals with diarrheal conditions have a greater risk for *Cryptosporidium* infection. The odd ratio (OR = 4.13) with 95% confidence interval (Lower = 2.11, Upper = 7.91) also shows an association of *Cryptosporidium* infection with diarrheal and non-diarrheal conditions, suggesting a statistically significant correlation.

### Prevalence of *Cryptosporidium* infection and monthly meteorological conditions

**Fig 3:**
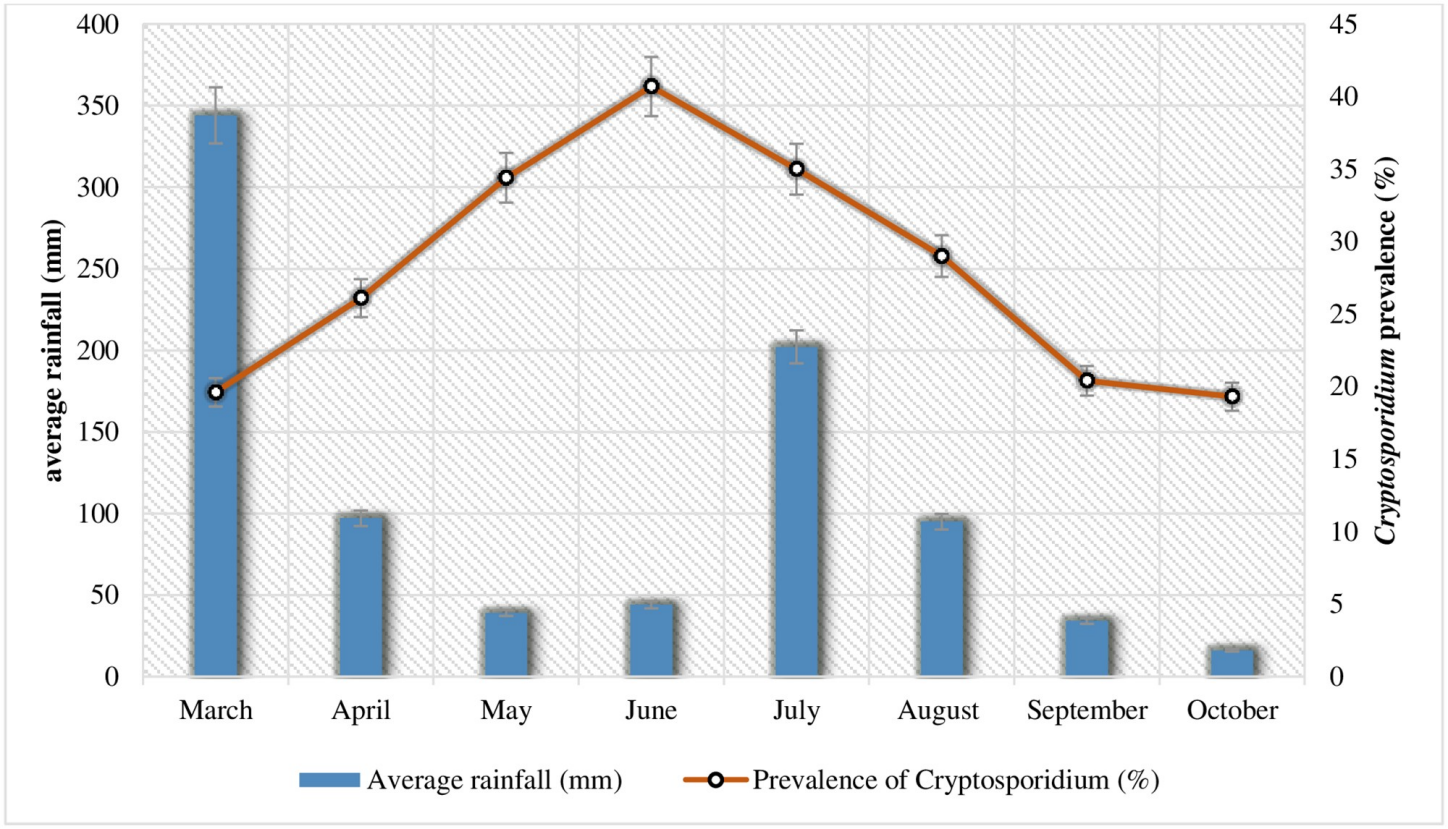
The graph shows the association between prevalence (%) of *Cryptosporidium* infection among studied participants (n = 425) and average rainfall (mm) recorded during the study.

The monthly metrological data shows the observed prevalence rate of *Cryptosporidium* infection in spring season (March 19.6%%, April 26.1%, May 34.4%) with average monthly temperature and rainfall of 19 °C/344 mm, 24 °C/97 mm and 31 °C/39 mm respectively. An increase in prevalence was observed in hot summer with rise in average temperature and decrease in rainfall during June when the prevalence was 40.7% (33 °C, 44 mm), July 35.0% (32 °C, 202 mm), August 29.0% (31 °C, 95 mm) and September 20.4% (30 °C, 34 mm). Contrarily, falling the monsoon (from October), a decrease in the prevalence was observed (19.3%) with reduction in average temperature and rainfall (26 °C, 16 mm) (Fig 3).

The statistical analysis for metrological data and the *Cryptosporidium* spp prevalence showed a significant correlation (OR = 2.01, 95% C.I = 1.28–3.13, *p*-value = 0.02) between the observed prevalence (Mean = 15.88, SD = ±8.8), average monthly temperature (Mean = 28.25, SD = ±4.83) and rainfall (Mean = 108.87, SD = ±111.88).

### Prevalence of *Cryptosporidium* infection in association to different water resources in the area

Statistically, the maximum prevalence rate (OR = 2.26, 95% C.I = 2.90–4.98, *p*-value = 0.001) was observed in individuals consuming contaminated water (drinking 43.01%, bathing 18.97%), which has been infected with *Cryptosporidium* spp. The results show a statistically significant association between *Cryptosporidium* spp prevalence with un-boiled water (OR = 2.32, 95% C.I = 1.42–3.74, *p*-value = 0.007). These results suggest that the prevalence of *Cryptosporidium* spp has a link to drinking the surface water (streams, lakes, rivers and springs) or recreational water contaminated with parasite oocysts (Table 3).

**Table 3:** The relationship between different water sources, quality and management with *Cryptosporidium* spp prevalence (n = 425 for each group).

Variables		+ve	%	Controls	%	OR	95% C.I	*p*-value
**Drinking Water source**								
Surface water	187	47	25.10%	140	74.80%	0.66	0.43–1.00	0.06
Ground water	238	80	33.60%	158	66.30%			
**Use of boil water**								
Yes	131	26	19.85%	105	80.15%	2.32	1.42–3.74	0.007*
No	304	111	36.51%	193	63.49%			
**Sewage water management**								
Poor	302	98	32.45%	204	67.55%	1.55	0.96–2.54	0.07
Satisfactory	123	29	23.58%	94	76.42%			
**Use of open source water**								
Drinking	271	83	43.01%	188	56.99%	2.26	2.90–4.98	0.001*
Bathing	154	44	18.97%	110	81.30%			

* Significant value < 0.05

The results of the consumption of ground and surface water suggest there is no statistically significant correlation (OR = 0.66, 95% C.I = 0.43–1.00, *p*-value = 0.06) between *Cryptosporidium* spp prevalence and water sources. There was no statically significant difference observed in sewage water management (OR = 1.55, 95% C.I = 0.96–2.54, *p*-value of 0.07) and prevalence of *Cryptosporidium* infection however, the small variation in values may be a possible factor for increased frequency of infection. These results confirmed that drinking or bathing in surface water may be a significant risk factor for *Cryptosporidium* spp infection. This was particularly the case for rivers that are not protected from human or animal fecal contamination. Surface waters, especially in rural areas mostly get muddy by cattle-farms manure or slurry used as fertilizer for crop farming and other agricultural purposes.

### Effect of economic status and parent’s education level on *Cryptosporidium* infection

Of all 127 participants found positive for *Cryptosporidium* spp the prevalence was influenced by economic variables in these analyses (OR = 1.61, 95% C.I = 1.04–2.52, *p*-value = 0.04). Individuals living in low-income families were also associated with increased odds of the prevalence. However, statistically there was no significant correlation found in parents level of education (1.49, 95% C.I = 0.94–2.38 and a *p*-value of 0.08).

Also, the prevalence of disease was not related to individuals working in agricultural farm (89/29.4%, Mean = 151.0, SD = ±87.6) and those not working in agricultural farms (38/30.8%, Mean = 61.50, SD = ±33.23). The odds ratio (0.93, 95% C.I = 0.59–1.96, *p*-value = 0.8) also shows a great variance suggesting that the *Cryptosporidium* spp prevalence is not statistically associated with these factors (Table 4).

**Table 4:** The prevalence (%) of *C*. *parvum* as a function of (A) economic status (B) parents level of education (C) working in agricultural farms.

Variables	N	+ve/%	Controls/%	OR	95% C.I	*p*-value
**Economic status**						
Poor	258	87/33.7%	171/66.2%	1.61	1.04–2.52	0.04*
Middle class	167	40/23.9%	127/76.0%			
**Parents level of education**						
Literate	107	39/36.4%	68/63.5%	1.49	0.94–2.38	0.08
Illiterate	318	88/27.6%	230/72.3%			
**Work in agricultural farm?**						
Yes	321	89/29.4%	213/70.5%	0.93	0.59–1.46	0.81
No	104	38/30.8%	85/69.1%			

*Significant value

### Contact with domestic animals and humans

These findings showed, that cases in contact with farmed or household animals and human are at significant risk with greater odds OR = 3.99 (95% C.I = 184.467) and OR = 2.49 (1.50–4.09) for sporadic prevalence of human cryptosporidiosis. The highest prevalence was observed in cases living with domestic animals (35.5%) and suspected human (39.0%). Statistically significant value was observed in those living with positive human (*p* = 0.001) and those living or came in contact with domestic animals (*p* = 0.002). These results indicate the zoonotic potential of *Cryptosporidium* spp in the study area and there may be a possibility that the high prevalence of infection is due to zoonotic transmission of the parasite (Table 5).

**Table 5:** The prevalence (%) of *Cryptosporidium* spp in response to variables for contact of individuals with suspected human or domestic animals (n = 425).

Variables	N	+ve/%	Controls	Mean	±SD	OR	95% C.I	*P*-value
**Living with other suspected human**								
Yes	241	94 (39.0%)	147 (49.4%)	120.0	±37.4	3.99	1.84–4.67	0.001*
No	184	33 (17.9%)	151 (50.6%)	92.0	±83.4			
**Living with domestic animals**								
Yes	287	102 (35.5%)	185 (64.4%)	143.0	±58.6	2.49	1.50–4.09	0.002*
No	138	25 (18.1%)	113 (81.8%)	69.0	±62.2			

*Statistically significant value

### Molecular characterization through PCR

**Fig 4:**
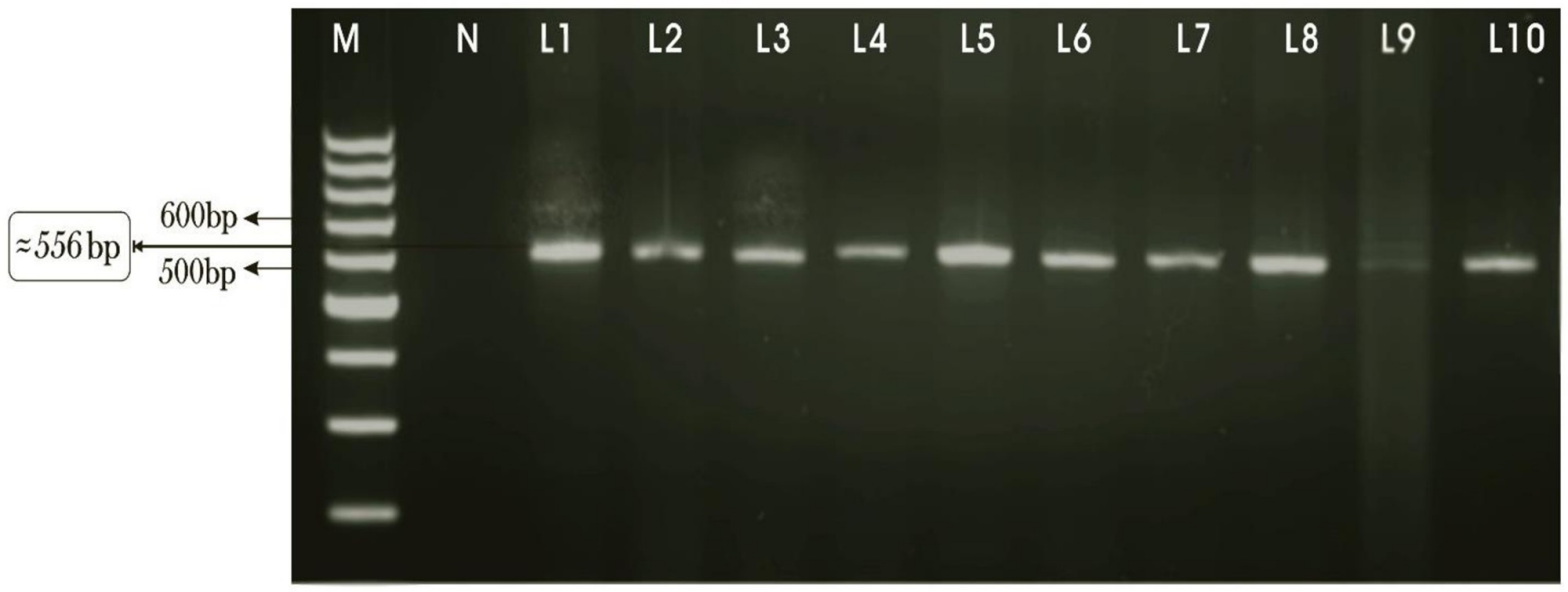
The PCR product (556 bp) after amplification of 18S rRNA gene of *C*. *parvum*. Lane-M: Marker (Fermentas, Germany), Lane-N: negative control and L1-L10: *C*. *parvum* positive samples.

The 71 samples out of all 127 mZN positive samples were observed to be positive by PCR method. On the other hand, 50 negative samples by mZN were tested by PCR reaction and 4 out of them were identified as positive (Fig 4).

## Discussion

In this study, the presence of *C*. *parvum* in young children (aged 3–10 years) was addressed for the first time in several geographic regions of District Buner by examining both asymptomatic and symptomatic children. *Cryptosporidium* oocysts were identified in 127 of all 425 (29.88%) collected samples through mZN staining technique. This prevalence is comparable to previous studies reported from Canada (18.02%), USA (21.2%), Australia (23.8%) and New Zealand (86.4%) [30–33]. Studies on human cryptosporidiosis from Bangladesh, India, Iraq, Kuwait, Tajikistan, UAE and Yemen also reported a high prevalence of 77%, 11.8%, 33.83%, 41.4%, 63.1%, 19.4% and 43.4% respectively [34–40]. Several factors such as age, gender, diarrheic and non-diarrheic conditions, personal hygiene, sewage water management, drinking or using untreated water, metrological conditions, contact with suspected animal or human and poor economic status of the families may play a key role in the current high prevalence of cryptosporidiosis.

The results revealed that there was no statistically significant difference between male (Mean = 17.64, SD = ±10.29) and female (Mean = 12.71, SD = ±4.51) in gender wise prevalence with a *p*-value of 0.38. Similarly, several studies have reported that there is no substantial association between *Cryptosporidium* infection and gender group [41–43].

The *Cryptosporidium* infection has been reported in people from age 3 days to 95 years however, statistical analysis show that the young kids are more vulnerable to *Cryptosporidium* infection [44]. The current findings suggest that children in the age group of less than 5 years were more exposed and infected (41.0%) with *Cryptosporidium* infection. Different environmental factors and low standard of personal cleanliness may have attributed to this higher infection of children. Similarly, some investigations from Malaysia show that most of infected individuals were children having less than four years of age [45]. This statement is an agreement with other described studies from Bangladesh, Nepal, Bhutan and Sri Lanka reporting infected cases from children below 3 years of age [46–48]. A similar study from Peshawar (Pakistan) also reported the infection in children less than 2 years of age [49].

Most of the positive cases were found that they were previously suffering from gastrointestinal symptoms and diarrhea. The two groups (diarrheic and non-diarrheic) were assessed and it was found that people with watery diarrhea (n = 330/425 (116/27.29%), Mean = 58, SD = ±35.36) are tended to have greater chance of being infected with *Cryptosporidium* spp (OR = 4.13, 95% CI = 2.11–7.91, *p*-value = 0.01). Similarly, several other findings from Kenya, Iran, Nigeria and Korea gives the impression of an agreement with these results which also reported that individuals with severe or persistent diarrhea were at greater risk for *Cryptosporidium* infection [50–52].

In present study, a significant association was observed between the season and prevalence of *Cryptosporidium* infection. The highest prevalence was observed during hot months of May, June and July (34.4%, 40.7% and 35.0% respectively). This is because, the people in these months mostly approach to take shower in surface water (rivers, streams, canals), and a higher temperatures (31 °C, 33°C and 32 °C) during these months are suitable for the viability of *Cryptosporidium* spp oocysts. Studies reported from India show that an increase in temperature and humidity causes an increase in the prevalence of *Cryptosporidium* infection [53, 54]. Supposedly, an increase rainfall during March (344 mm) and July (202 mm) may be responsible factor for the dispersal of infective oocysts eventually into the surface water (rivers, canals, streams). Furthermore, the rivers, streams and canals of the study area are used for irrigation purposes, cattle feeding and at the same time as drinking water source for local residents which increase the chances of *Cryptosporidium* infection.

These findings also suggest that individuals consuming surface water (for drinking or bathing purposes) were at risk for infection. Most of the participants were from villages and rural areas where birds, cats and dogs are commonly wandering freely, which may be a route for subsequent zoonotic spreading of oocysts, contaminating the soil and water with their feces. Cows, buffalos and other domestic animals are also seen drinking and bathing in the surface water (rivers, streams and canals) along with children. A study from three Districts of KP Pakistan showed that *Cryptosporidium* infection was prevalent (19.5%) in surface water as compared to other water-borne parasites [55]. In lowland UK, the *Cryptosporidium* infection was pre-dominant in livestock and deer samples, suggesting a significant risk to surface water quality and public health [56]. In 2002, a high prevalence (66%) of *Cryptosporidium* infection in surface waters on a coastal farm in England was reported, where *Cryptosporidium* spp oocysts were being spread by at least one livestock or wild animal inhabitants [57]. Similarly, other studies from North West Wales and Scotland evidenced that wildlife contributes to the oocyst counts in surface waters [58, 59]. A study from China reported that the drinking of surface water is the main cause of the *Cryptosporidium* exposure route and infection [60]. During present study, the human feces were often found near surface water and houses and in some towns the sewage and toilets waste water were freely flowing to the surface water sources which is a concern for possible water-borne transmission of *Cryptosporidium* infection. Even though, *Cryptosporidium* spp oocysts are resistant to many disinfectants, but it can be inactivated by heating water up-to 62 °C [61]. Likewise, the WHO guidelines for drinking water quality also suggest that *Cryptosporidium* spp oocysts are non-viable and can be inactivated at 60 °C. There was a very low risk of infection to those who usually use boiled water for drinking. In addition, present study also revealed that individuals who were consuming un-boiled surface water were at greater risk (*p*-value = 0.007) for *Cryptosporidium* infection, while those who used boiled water for consumption were found protective against infection.

The findings of our study are in agreement that families with low economic status are at significant risk (*p*-value = 0.04) for *Cryptosporidium* infection in the study area. Statistically, there was no significant association observed among the parent level of education (*p*-value = 0.08) and those working in agricultural farms (*p*-value = 0.81). Comparable studies from USA and Brazil reported that *Cryptosporidium* infection is the highest risk for people living in families with poor food adequacy [62, 63]. The food insufficiency may push the families to access food products from places where food safety and hygiene is unconsidered, such as at open street marketplaces [64].

The direct contact with infected animals is supposed to be an important mode of transmission because adult cattle are considered as major source for prolong shedding of oocysts [65]. A study conducted on dairy farm workers in Zambia showed a significant prevalence (22.5%) with overall prevalence of 34% in infected animals [66]. In some studies contact with contaminated feces spread by birds and insects, through sneezes and exposed hand lesions have been linked with outbreaks [67]. The native farmers who use animal dung and human waste as an organic fertilizers or those who have a close and frequent contact with domestic animals are also at risk for *Cryptosporidium* infection [68]. The direct contact with a child in a child-care programme or with diapers may also contribute in spreading of this parasite to other individuals [69]. Our results show that among all 425 cases, 241 respondents living with a person who was positive for *Cryptosporidium* infection had greater risk to acquire the infection (OR = 3.99; 95% CI 1.84–4.67; *p* = 0.001). Similarly, 287 peoples that were living with domestic animals (cow, sheep, buffalo, goat etc.), were tended to have a greater odds of infection (OR = 2.49; 95% CI 1.50–4.09; *p* = 0.002).

The samples identified as positive by mZN staining for *Cryptosporidium* spp oocysts were effectively proved positive by PCR analysis which suggest the use of molecular approaches in combination with microscopic techniques for overcoming the diagnosis difficulties [70]. The mZN staining method do not differentiate between the genotypes and sub-types which leave the researcher without specific information about the species or genotypes. Present study endorse that PCR assay is highly sensitive and specific and should be used for the screening of *Cryptosporidium* spp. However, in unindustrialized countries and under field conditions, where limited resources do not allow the application of PCR assay, the staining methods are recommended for routine-based detection and diagnosis of *Cryptosporidium* spp.

## Conclusions

The current study authenticates the fact that *Cryptosporidium* spp infection is predominant in children and young individuals (aged 3–10 years) in different areas of District Buner. The cryptosporidiosis remains undiagnosed due to the lack of microscopic examination of fecal samples in this region. Molecular studies are essential to precisely understand the mode of transmission, risk factors and the genetic diversity of *Cryptosporidium* spp in humans and domestic animals across the country. These investigations will provide a valuable source for the prognostic epidemiology of *Cryptosporidium* infection.

## Supporting information

S1 File Questionnaire for data collection from participants. (PDF)
