# Supplementary material for: Evaluation of prevalence and risk factors associated with Cryptosporidium infection in rural population of district Buner, Pakistan
Source: PLoS One. 2019 Jan 2;14(1):e0209188. doi: 10.1371/journal.pone.0209188 (PMC6314602; doi:10.1371/journal.pone.0209188)
Supplement: S1 File — (PDF) [file pone.0209188.s001.pdf]

DEPARTMENT OF ZOOLOGY

ABDUL WALI KHAN UNIVERSITY MARDAN, KP PAKISTAN

Questionnaire for Study participants (suspected individuals) data collection

Tick (√) the relevant box   Date: ______/______/_____

**Name:**		**1. Age:**	☐ ≤ 5 years ☐ > 5 years
**2. Gender:**	☐ Male Female ☐	**3. Location:**	☐ Hospital ☐ Village
**4. Symptoms present**		☐ Diarrheic	☐ non-diarrheic
**5. Source of drinking water**		☐ Ground water	☐ Surface water
**6. Use boiled water**		☐ Yes	☐ No
**7. Sewage water management**		☐ Poor	☐ Satisfactary
**8. Use of open source water**		☐ Drinking	☐ Bathing

***Socio-economic and educational variables of the infected individual's families**		
**9. Parents level of education**	☐ Illiterate	☐ Littrate
**10. Socio-economic conditions**	☐ Poor	☐ Middle class
**11. Working in agricultral land**	☐ Yes	☐ No
***Contact with other household infected animals or humans**		
**12. Living with other +ve person**	☐ Yes	☐ No
**13. Living with household animals**	☐ Yes	☐ No
